# Impact of asymptomatic *Plasmodium falciparum* parasitemia on the imunohematological indices among school children and adolescents in a rural area highly endemic for Malaria in southern Mozambique

**DOI:** 10.1186/1471-2334-13-244

**Published:** 2013-05-27

**Authors:** Eduardo Samo Gudo, António Prista, Ilesh V Jani

**Affiliations:** 1 Instituto Nacional de Saúde, Maputo, Mozambique; 2 Faculty of Physical Education and Sport Sciences, Universidade Pedagógica, Maputo, Mozambique

## Abstract

**Background:**

Asymptomatic *Plasmodium falciparum* parasitemia (APFP) has been reported to be highly prevalent in Sub-Saharan Africa, a region heavily burdened by malaria, yet, the impact of APFP on the immunological reference values have not yet been established. This study was aimed at i) determine the prevalence of APFP in children and adolescents living in a region highly endemic for malaria in southern Mozambique and its impact on the immuno-hematological indices and ii) determine the factors independently associated with APFP.

**Methods:**

A cross sectional study was conducted in a rural area highly endemic for Malaria in southern Mozambique during the dry season. Apparently healthy children and adolescents were selected for the study.

**Results:**

Blood samples were collected from 348 participants. *Plasmodium falciparum* was detected in 56.5% (194/343) of study subjects. APFP was more frequent in males and was associated with lower values of hemoglobin and platelets measurements. Parasitized and not parasitized individuals were similar in terms of lymphocyte counts, CD4+ and CD8+ T cells counts. Platelet count was the parameter with strongest association with APFP (OR: 0.991, p= 0.000) in children and its performance in guiding clinical suspicion was moderate (AUC: 0.70, p=0.000). Contrarily, in adolescents, the predictive value of platelets counts was low (AUC: 0.55).

**Conclusion:**

Overall, our finding demonstrated that APFP is highly prevalent in regions endemic for malaria in southern Mozambique and was associated with lower hematological parameters but unaltered lymphocyte counts, CD4+ and CD8+ T cells counts. Platelets count was of moderate performance in guiding clinical suspicion of APFP in children but not in adolescents.

## Background

Asymptomatic *Plasmodium falciparum* parasitemia (APFP), defined as the presence of malaria parasites in peripheral blood in absence of symptoms, has been described to be prevalent in regions highly endemic for malaria. Previous studies conducted in Mozambique and other sub-Saharan African countries showed that a large proportion of individuals living in malaria endemic regions harbour asymptomatic malaria disease [1-5].

The spectrum of clinical consequences associated with APFP is not fully understood, but it is well known that APFP causes several haematological alterations, with anaemia and thrombocytopaenia being the most prominent [6]. However, little is known regarding the impact of APFP on immunological parameters. We hypothesize that APFP represents a major factor driving and influencing the immunological characteristics among apparently healthy people living in malaria endemic areas in Southern Africa.

Immunological measurements, including CD4+ and CD8 T+ cells counts, play a key role in the diagnosis, prognosis and monitoring of a wide variety of diseases [7-11]. The role of CD4+ T cells measurements has been renewed in recent years in sub-Saharan Africa as a result of the HIV/AIDS pandemic. In this region, CD4+ T cells counting is one of the most important laboratory parameter in HIV Medicine, as it is used to stage HIV disease, to decide when to start Highly Active Antiretroviral Therapy (HAART) or prophylaxis against opportunistic infections, to assess HAART efficacy and to perform AIDS surveillance [12,13].

A previous report from our group demonstrated that children living in southern Mozambique presented almost the double amount of CD4+ T cells counts when compared to children from western countries [14], raising the concern that international cut-offs used for laboratory monitoring of HIV and HAART might not be adequate for children and adolescents living in sub-Saharan countries, particularly in places where malaria is highly endemic. The extent to which APFP contributes to these differences remains unknown.

In a time when HAART is being rapidly scaled-up globally, there is a need to determine the impact of APFP on the profile of immunological parameters, particularly on the profile of CD4+ T cells counts in regions where both malaria and HIV are highly prevalent.

We conducted this study aiming at determining: i) the prevalence of APFP in a region highly endemic for malaria, ii) the impact APFP on the immuno-hematological indices, and iii) the factors independently associated with APFP.

## Methods

### Study setting

The study was conducted in Calanga, a small village on the eastern coast of southern Mozambique. The houses are precarious with neither piped water, nor electric power supply. Calanga has an estimated population of 9451 inhabitants and is located in an area with high endemicity and transmissibility of malaria. The raining season starts from November through April. The main occupation of the population is subsistence-level farming and cattle grazing. A smaller proportion of the population, mainly adults, works as seasonal sugar cane cutters or fishermen. At the time of our study, only one health facility in Segera, a small community that is part of the village, was available to serve all of the surrounding area.

### Study sample

Between August and September 2005, we consecutively enrolled individuals attending 1st (1st - 5th grades) and 2nd (6th - 7th grades) levels of the primary education in Calanga. Consent to participate in the study was obtained through parents or guardians. Before enrollment, information about study objectives and procedures was provided and explained to local authorities, students and their guardians in the local language. Study staff read and explained the consent form in the local language for illiterate parents or guardians. After consent was signed, students were submitted to a clinical evaluation by a physician. Individuals presenting any sign or symptoms of disease such as fever were excluded from the study. The study was approved by Mozambique’s National Health Bioethics Committee. This study was part of a larger project entitled “Human Biological Variability: Implication for Physical Education, Sports, Preventive medicine and Public Health” [15].

### Specimen collection

Venous blood (10 ml) was collected in all subjects who consented to participate in the study. Blood was collected from the cubital fossa into a 5 ml K3EDTA tube and a 5 ml Serum Separation Tube (both from BD Vacutainer, USA). Specimens were delivered to the laboratory within four hours of collection.

### Laboratory assays

#### Blood smear microscopy

Upon delivery of samples at the laboratory, a thick blood smear was mounted from anticoagulated whole blood. All blood smears were stained using the Giemsa protocol and screened for *Plasmodium falciparum*, *P*. malarie, *P. ovale* and *P. vivax* using light microscopy. Parasite density was estimated by means of a semi-quantitative scale [16].

#### Hematology

Hematology parameters, including white blood cell counts and differentials, were determined using an automated five-part differential hematology analyzer (Sysmex SF 3000, Japan). All samples were processed within six hours of collection.

#### Immunophenotyping for T cell subsets

Immunophenotyping was performed on fresh whole blood using a FACSCalibur^TM^ flow cytometer (Becton Dickinson, USA). MultiTEST reagents, TruCOUNT tubes and MultiSet software (all from Becton Dickinson, USA) were employed in a lyse-no wash protocol to determine absolute and percent values for T cell subsets (CD4+ T cells, CD8+ T cells and CD4/CD8 ratio) [17].

### Statistical analysis

Data was analyzed using the statistics package STATA 9.0 (College Station, Texas: StataCorp, USA, 2005). The Mann Whitney test was used to compare the study groups regarding numerical variables. Associations between categorical variables were determined using the Pearson Chi-square test.

Logistic regression analysis was employed to determine the variables independently associated with APFP, controlling for confounders. The main confounders used for multivariate analyses were: gender, hematocrit, hemoglobin concentration and platelet count. The analysis was built using backwards stepwise method and Log Likelihood Ratio Test. For this purpose, all variables with a *P-*value less than 0.25 on univariate analysis were included in the initial multivariate model. A *P* value ≤ 0.05 was considered of statistical significance on the final model.

Receiver Operational Characteristics (ROC) curve analysis, sensitivity, specificity, and predictive values (positive and negative) were used to determine the performance of platelets count in predicting APFP. The gold standard method for APFP diagnosis relied on blood smear. ROC curve analysis was also used to define the best cut-off point of platelet count to predict APFP. The Area Under the ROC curve (AUC) was computed by the trapezoidal rule.

For the purpose of comparison of immuno-hematological values, we stratified the participants by age, using the cut-off of 12 years of age. Those younger than 12 years were denoted as children and those older than 12 years were denoted as adolescents.

## Results

### Baseline characteristics of study subjects and prevalence of asymptomatic Plasmodium falciparum

Consent to participate and blood samples were obtained from 348 subjects. Of these, 55% (190/348) were male and the median age of study subjects was 11 years old (IQR: 8–13). *P. falciparum* was the dominant species of Plasmodium in this study and was evidenced in 195 individuals, giving an overall prevalence of APFP of 56.5% (95% IC 51.3% - 62.8%). *P. malarie* infection was evidenced in 7 samples (2.6%). No sample was positive for either *P. ovale* or *P. vivax*. For the purposes of this study, the terminology parasitized denotes infection by *P. falciparum*.

Table 1 depicts the distribution of APFP by age, gender and basic anthropometric measures. Compared to adolescents, children presented a significantly higher prevalence of APFP (61.5% *versus* 47.3%, p=0.007). In both children and adolescents, the prevalence of APFP was higher in boys compared to girls (71.9% *versus* 56.3% for children and 56.0% *versus* 43.5% for adolescents), and these differences were statistically significant for children (p=0.044) but not for adolescents (p= 0.091).

**Table 1:** General characteristics of study subjects stratified by presence of APFP

**Age (years)**						
**Parameter**		**5 - 11**			**12 - 20**	
	**Parasitized (n=116)**	**Non-parasitized (n=68)**	p-value*	**Parasitized (n=78)**	**Non-parasitized (n=81)**	p-value*
**Gender**						
Male	59/82 (71.9%)	23/82 (28.1%)		56/100 (56.0%)	44/100 (44.0%)	
Female	40/71 (56.3%)	31/71 (43.7%)	**0.044**	37/85 (43.5%)	48/85 (56.5%)	0.091
**Height (cm)**						
Median	121	121		145	152	
IQR	115-128	115-127	0.974	138-154	141-158	**0.036**
**Weight (Kg)**						
Median	21.6	22.3		36.3	40.1	
IQR	19.9-25.6	20.0-25.0	0.693	30.4-43.7	32.5-46.9	**0.042**
**BMI (Kg/m**^**2**^**)**						
Median	15.4	15.4		16.8	17.6	
IQR	14.5-16.3	14.6-16.0	0.952	15.6-18.1	16.4-18.8	**0.020**

* Mann–Whitney test.

Parasitized adolescents presented lower height (parasitized 145 cm *versus* non-parasitized 152 cm, p=0.036), weight (parasitized 36.3 Kg versus non-parasitized 40.1 Kg, p=0.042) and BMI (parasitized 16.8 Kg/m^2^*versus* non-parasitized 17.3 Kg/m^2^, p= 0.020) (see Table 1). Parasitized and non-parasitized children were similar regarding weight, height and BMI (see Table 1).

### Influence of plasmodium falciparum in immuno-hematological characteristics

As shown in Table 2, parasitized children but not adolescents presented significantly lower hemoglobin levels (11.1 g/dL *versus* 11.6 g/dL, p=0.01 for children and 11.8 g/dL *versus* 12.2 g/dL, p= 0.07 for adolescents). In addition, both children and adolescents with APFP presented significantly lower hematocrit levels (32.2% *versus* 33.6%, p=0.01 for children and 34.3% *versus* 35.4%, p= 0.04 for adolescents). Platelet count in parasitized children was lower than in non-parasitized children (p=0.00), but this difference was not observed in adolescents (p= 0.283).

**Table 2:** **Median and 90**^**th **^**percentile range for Hgb, MCV, Htc, MCHC and PLT indices: stratification by age and presence of *P. falciparum *infection**

**Age (years)**						
**Parameter**	**5 - 11**			**12 - 20**		
	**Parasitized (n=116)**	**Non-parasitized (n=68)**	p-value*	**Parasitized (n=78)**	**Non-parasitized (n=81)**	p-value*
**Hgb (g/dL)**						
Median	11.1	11.6		11.8	12.2	
p5-p95	8.9-12.6	9.1-12.6	**0.01**	10.1-13.8	10.0-13.9	0.07
**MCV (fL)**						
Median	79.2	79.0		79.7	80.9	
p5-p95	68.4-86.1	67.8-88.5	0.805	70.5-88.3	70.5-89.6	0.130
**Htc (%)**						
Median	32.2	33.6		34.3	35.3	
p5-p95	26.5-36.3	27.8-36.2	**0.01**	29.2-39.1	29.9-40.4	**0.04**
**MCHC (g/dL)**						
Median	34.3	34.8		34.5	34.5	
p5-p95	32.3-36.6	32.1-36.2	0.458	31.6-36.8	32.0-36.8	0.923
**PLT (10**^**3**^**/mm**^**3**^**)**						
Median	241	282	**0.000**	223	228	0.287
p5-p95	133-358	167-397		141-358	110-327	

* Mann–Whitney test.

Parasitized and non-parasitized children and adolescents were comparable regarding Mean Corpuscular Hemoglobin Concentration (MCHC) levels (p=0.458 for children and p=0.923 for adolescents) Regarding total leukocytes and its subsets counts, there were no differences between parasitized and non-parasitized children and adolescents, except for CD8+ T cells percentage count in adolescents for which parasitized individuals presented higher counts (25.8% versus 23.6%, p=0.035, see Table 3).

**Table 3:** **Median and 90**^**th **^**percentile range for WBC, LY, and lymphocytes subsets indices: stratification by age and presence of *P. falciparum***

**Age (years)**						
**Parameter**		**5 - 11**			**12 - 20**	
	**Parasitized (n=116)**	**Non-parasitized (n=68)**	p-value*	**Parasitized (n=78)**	**Non-parasitized (n=81)**	p-value*
**Total leukocytes count (10**^**3 **^**cells /mm**^**3**^**)**						
Median	7.1	6.9		6.4	6.2	
p5 – p95	4.4-11.6	4.7-11.4	0.985	4.5-9.0	4.3-9.7	0.704
**Total lymphocytes count (10**^**3 **^**cells /mm**^**3**^**)**						
Median	3.8	4.0		3.1	3.3	
p5 – p95	1.8-7.7	2.4-7.1	0.608	2.0-5.1	1.8-6.1	0.410
**CD3+ cells (10**^**3 **^**cells /mm**^**3**^**)**						
Median	2.7	2.8		2.3	2.3	
p5 – p95	1.3-5.6	1.7-5.3	0.575	1.5-3.5	1.3-4.5	0.553
**T CD4+ cells (cells /mm**^**3**^**)**						
Median	1588	1664		1306	1412	
p5 – p95	728-3414	873-2831	0.980	829-2095	820-2440	0.195
**T CD8+ cells (cells /mm**^**3**^**)**						
Median	852	942		788	789	
p5 – p95	383-2042	412-1971	0.287	454-1695	378-1622	0.855
**T CD4+ cells (%)**						
Median	42.4	41.7		42.1	42.9	
p5 – p95	31.7-53.1	30.3-50.5	0.420	28.4-53.2	33.9-53.4	0.485
**T CD8+ cells (%)**						
Median	22.8	23.8		25.8	23.6	
p5 – p95	15.1-32.3	16.3-34.1	0.254	16.5-35.8	16.5-30.3	**0.035**
**T CD4+/CD8+ ratio**						
Median	1.8	1.7	0.264	1.6	1.9	0.072
p5 – p95	1.1-3.3	0.7-3.0		0.8-3.2	1.2-2.9	

* Mann–Whitney test.

### Relationship between anemia, age and asymptomatic plasmodium falciparum infection

Table 4 demonstrates the distribution of anemia in the study group stratified by age and APFP. Anemia was more prevalent in children compared to adolescents. Moreover, the proportion of anemic individuals was higher in parasitized children (67.2% *versus* 44.1%, for parasitized and non-parasitized) and adolescents (57.7% *versus* 44.5% for parasitized and non-parasitized). This difference was statistically significant in children (p=0.002) but not in adolescents (p=0.095) (see Table 4).

**Table 4:** **Prevalence of anemia in the study subjects: stratification by age and presence of *P. falciparum***

**Characteristics**	**Definition of anemia, (g/dL) **^**a**^	**Anemic % (n)**	**p-value**
***Plasmodium falciparum *status , 5.0 - 11.9 years old**	**<11.5**		
Parasitized		67.2 (78/116)	
Non parasitized		44.1 (30/68)	**0.002**
***Plasmodium falciparum *status , 12.0 - 14.9 years old**	**<12.0**		
Parasitized		57.7 (45/78)	
Non parasitized		44.4 (36/81)	0.095

^**a**^ – Source: WHO [18].

### Predictors of asymptomatic plasmodium falciparum infection in children younger than 12 years

Since the most remarkable consequences of APFP were observed in children, we investigated if any of clinical, demographic or laboratory parameters could be used as a predictor for APFP and guide clinicians in the management of APFP. As seen before, gender and hematological differences between parasitized and non-parasitized individuals were stronger in children. The final model for logistic regression analysis comprised platelets count, gender and hemoglobin. As the platelets count was the variable with the strongest association with APFP in children (data not shown), subsequent investigation of prediction of APFP in children was focused on platelets counts.

**Figure 1:**
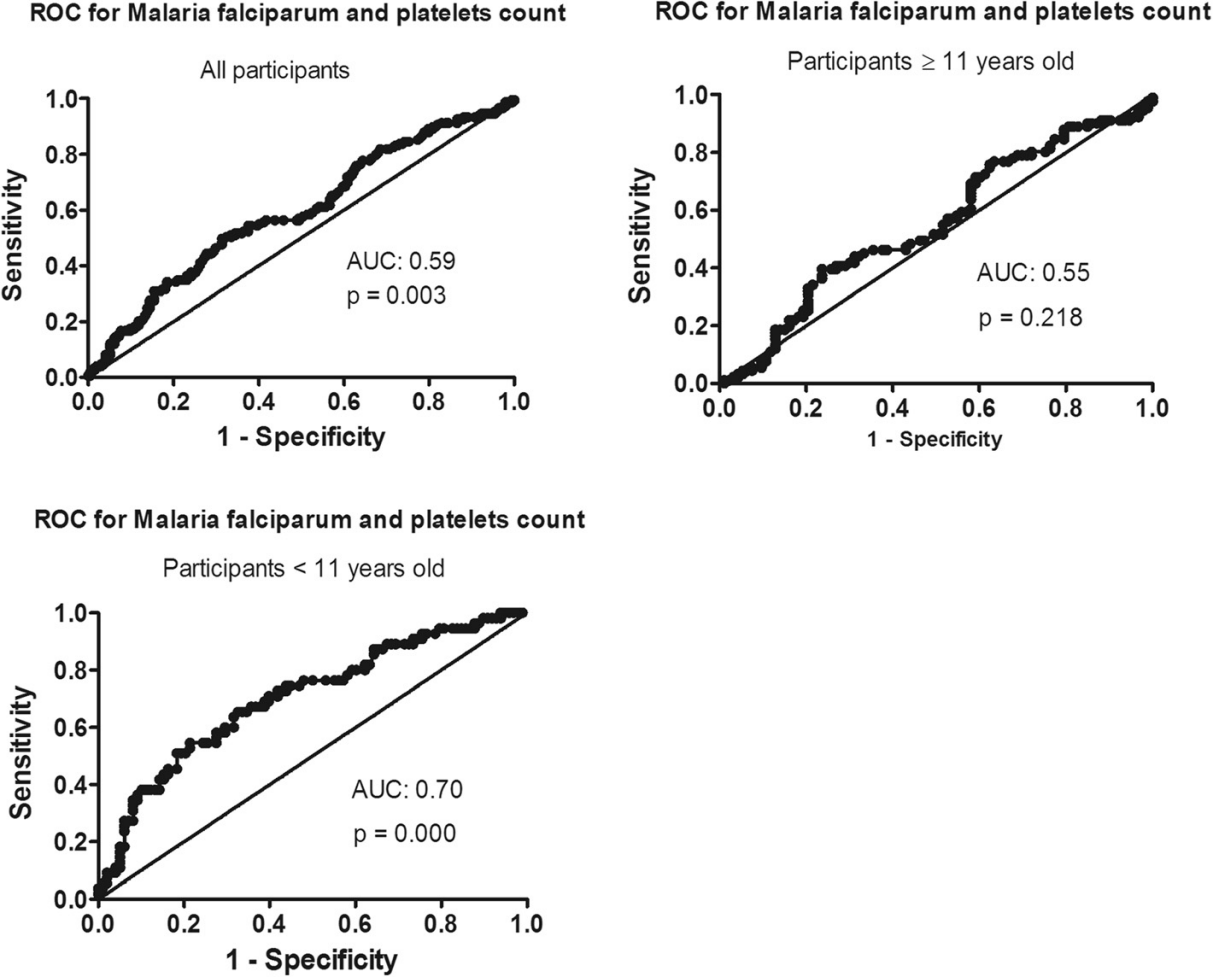
**Receiver Operational Curve analysis of platelets counts to detect asymptomatic *P falciparum *infection in children and adolescents attending primary school in a malaria endemic village in Southern Mozambique.**

The performance of platelets counts for predicting APFP was higher in children (AUC: 0.70, p=0.00, Figure 1A), compared to adolescents (AUC: 0.59, p=0.03, Figure 1B). ROC analysis also demonstrated that the performance of platelets counts for predicting APFP when using unstratified data was very poor (AUC: 0.55, p=0.218, Figure 1C). Using ROC analysis, the best cut-off point of platelets count to guide the clinical suspicion of APFP in children was set at 272 cells/mm^3^. At this cut-off, the sensitivity, specificity, positive predictive value (PPV) and negative predictive value (NPV) were 60.2%, 70.4%, 78.7% and 49.4%, respectively.

## Discussion

In sub-Saharan Africa, a region heavily burdened by malaria and where APFP has been reported to be highly prevalent [1-3], little information exists regarding the impact of APFP on the immunological profile, particularly among children and adolescents, the groups mostly affected by malaria. The few studies conducted addressing APFP, focused mostly on haematological parameters, particularly on haemoglobin and platelets measurements [6]. Our results demonstrated that more than half of apparently healthy school children and adolescents in a malaria endemic rural area in Southern Mozambique presented parasitemia. This finding corroborates previous published data from others sub-Saharan Africa countries [1,6] and confirms our previous hypothesis that the prevalence of APFP in our settings is high. In addition, the vast majority of APFP were attributed to *P. falciparum* (195/348, 56.5%), with only a minor proportion of samples positive for *P. malarie* (7/348, 2.6%) and no circulation of either *P. ovale* or *P. vivax.* This was an expected finding since *P. falciparum* represents the main cause of malaria in our setting. The real prevalence of APFP might be even higher in Calanga as microscopic diagnosis of APFP underestimates the real prevalence of parasites when compared to molecular diagnosis [1,19].

The prevalence of APFP in boys was higher compared to girls. This result corroborates findings from previous studies conducted in Kenya [1,19]. The reasons behind this fact remain to be clarified, but we can speculate that this would be associated with biological variability, such as hormonal differences, differences in exposure to mosquito bites and difference in terms of cellular and humoral immune responses [20,21] Besides these gender differences, we showed that APFP decreased with age. These findings are similar with previous reports reflecting acquisition of immunity to *P. falciparum* in older age [1,19].

Regarding hematological findings, results from this study demonstrated an association between APFP and anemia and thrombocytopenia, in line with data from previous reports [6]. Although our data suggests that APFP would be a leading cause of anemia and thrombocytopenia in children living in Calanga, other causes of anaemia and thrombocytopenia cannot be excluded. In fact a recent countrywide survey demonstrated that soil transmitted parasites are highly frequent among schoolchildren in Mozambique [22], suggesting that intestinal parasites could contribute to anemia in this age group. In our study we were not able to gather information on the prevalence of intestinal parasites among the study subjects.

Apart from the hematological parameters, the impact of APFP on immunological indices has not been previously studied in-depth and published literature in this regard is scarce [6]. The understanding of whether or not APFP is associated with lower or higher CD4+ T cells counts is of utmost importance for the management of patients in the context of HIV/AIDS. A previous report from our group demonstrated that children living in Calanga presented almost the double amount of CD4+ T cells counts compared to children from western countries [14]. However, the extent to which APFP contributes to these differences has not been previously addressed. Our study was the first one to investigate whether APFP would influence the CD4+ T cell counts. We demonstrated that parasitized and non-parasitized children and adolescents were similar in terms of quantitative immunologic parameters. We were not able to investigate whether APFP could modify the functionality of cells of the immune system.

 In addition to the impact on the immunohematological profile, the presence of APFP is of additional concern as individuals with APFP: i) represent an important reservoir of transmissible parasites [19,23], ii) are at a higher risk of developing febrile malaria disease [2], and iii) have a very low rate of spontaneous clearance without treatment [2]. In this context, we developed an algorithm to guide clinicians in the investigation of APFP in settings where malaria is highly endemic. To our knowledge, this represents the first attempt to investigate predictors of APFP in children and adolescents. Previous reports demonstrated a strong predicting value of thrombocytopenia for guiding the diagnosis of febrile malaria [24-26]. However, it has never been investigated whether these results are applicable to afebrile malaria. Results from this study demonstrated that platelets count was strongly associated with APFP in children, but not in adolescents. The lower prediction value of platelets in guiding the suspicion of *P. falciparum* infection in afebrile individuals when compared to febrile individuals, suggests that the modifications induced by *P. falciparum* on the life cycle of platelets are of lower magnitude in asymptomatic individuals. This fact may be related with the acquisition of immunity.

## Conclusion

APFP is highly prevalent in regions highly endemic for malaria in Southern Mozambique and was associated with lower haematological values in school children but less in school adolescents. APFP represents an important cause of anaemia and thrombocytopenia in school children and adolescents in our settings. Contrarily, APFP was not associated with modifications on the immunological measurements commonly used to monitor HIV infection, such us CD4+ and CD8+ T cells counts. Thrombocytopenia was of moderate value in guiding clinical suspicion of APFP in children but not in adolescents.

## Competing interests

Authors declare they have no competing interests

## Authors’ contributions

ESG participated in the study design, data collection, data analysis, and writing the manuscript. AP participated in the study design, data collection, data analysis and writing the manuscript. IVJ participated in the study design, data collection, data analysis and writing the manuscript. All authors have read and approved the final manuscript.

## Pre-publication history

The pre-publication history for this paper can be accessed here:

http://www.biomedcentral.com/1471-2334/13/244/prepub
